# Improving the Effectiveness of Conservative Treatment of Idiopathic Scoliosis Through Active Parental Participation During Inpatient Rehabilitation

**DOI:** 10.3390/healthcare13131551

**Published:** 2025-06-29

**Authors:** Marianna Białek, Justyna Pękala, Ewelina Białek-Kucharska, Małgorzata Poczynek, Paulina Poświata, Tomasz Kotwicki

**Affiliations:** 1 FITS Method, 59-411 Paszowice, Poland; vewelinav@o2.pl; 2 Kinetik, 59-400 Jawor, Poland; biuro.kinetikjp@gmail.com; 3 FizMal, 59-300 Lubin, Poland; gosallegro@gmail.com; 4 FizjoUśmiech, 01-803 Warszawa, Poland; fizjousmiech@gmail.com; 5 Department of Spine Disorders and Pediatric Orthopedics, University of Medical Sciences, 61-545 Poznan, Poland; kotwicki@ump.edu.pl

**Keywords:** scoliosis therapy, exercise therapy, adolescent idiopathic scoliosis, FITS method, physiotherapeutic scoliosis-specific exercises (PSSEs), Anterior Trunk Symmetry Index (ATSI), Posterior Trunk Symmetry Index (POTSI), angle of trunk rotation (ATR), spinal deformity

## Abstract

Background/Objectives: This is a study of adolescents with idiopathic scoliosis (IS), treated by the Functional Individual Therapy of Scoliosis (FITS) method. The hypothesis was that active parental involvement in the treatment process enhances the outcomes of therapy. Materials and Methods: A total of 208 adolescent girls with IS were examined and divided into two groups. Only in Group I were the parents present. Trunk morphology before and after was evaluated by measuring change of the Anterior Trunk Symmetry Index (ATSI), Posterior Trunk Symmetry Index (POTSI), Posterior Trunk Symmetry Index in Correction (POTSI COR), and the angle of trunk rotation (ATR). Results: Statistically significant improvements in ATSI values were observed in both groups (Group I: *p* < 0.001; Group II: *p* = 0.001). POTSI values showed improvement only in Group I (*p* < 0.001). Similarly, POTSI COR values improved significantly in Group I (*p* < 0.001). ATR improved significantly better in Group I. Conclusion and Significance: Children with IS who underwent specific physiotherapy demonstrated better outcomes when their parents actively participated in the therapy.

## 1. Introduction

Scoliosis is a structural, three-dimensional deformity of the spine (visible on anteroposterior and lateral X-rays) and the trunk, evident during clinical examination [1]. One of the hypotheses about adolescent idiopathic scoliosis is represented by vicious cycles, which means that a spinal curve creates asymmetrical loading on the spine. This uneven pressure promotes asymmetrical vertebral growth, which then intensifies the curve. The cycle perpetuates itself, with the altered growth contributing to a more pronounced curve and increased asymmetrical loading. [2] The diagnosis of scoliosis is confirmed based on an X-ray when the Cobb angle is measured to be ≥10°. The condition tends to progress during consecutive growth spurts, particularly in puberty when skeletal maturity is 0–1 (Risser test) [1]. Treatment options, i.e., conservative (physiotherapy/physiotherapy with bracing) or surgical, depend on factors such as family history, degree of hypermobility, curvature angle, stage of skeletal maturity, and onset of menstruation in girls. In conservative treatment, according to the guidelines of the International Society on Scoliosis Orthopedic and Rehabilitation Treatment (SOSORT) [1], the current approach is represented by specific methods, such as physiotherapeutic scoliosis-specific exercises (PSSEs) [3,4,5,6,7].

The Functional Individual Therapy of Scoliosis (FITS) method is endorsed by the SOSORT Society as one of the PSSEs [3,8]. The method was introduced by Białek and Mhango in 2004 [9]. It comprises preparation for corrections using soft tissue techniques, education on a specific 3D self-corrective movement, and stabilization of the corrected position. FITS is a method that provides exercises that are well tailored to the needs of patients with scoliosis and is aimed at improving clinical symptoms, reducing the progression of the condition or Cobb angle, and enhancing the child’s physical abilities and overall fitness [10]. Scoliosis can be divided according to the size of the Cobb angle, the number of curves, and the directions of curvature. The largest groups of the two-curve scoliosis are: right-sided thoracic and left-sided thoracolumbar. Each of these types of two-curve scoliosis is unique; they differ from the other in the size of the curvature angle, in the size of how the two curves compare to each other, by the place of the greatest rotation of the torso, by the position of the pelvis and shoulder line, by the appearance of the structural compensation (e.g., third curve), or by functional compensation (only in soft tissues). Patients with scoliosis will also differ from each other by the ability to stabilize the lower torso, proprioception, and even by the level of involvement in the treatment process. Taking these factors into account, it is necessary to approach each patient individually, adjusting the therapy to the current clinical and radiological conditions and the patient’s capabilities. It is also necessary to determine the goals that are achievable: to stop progression or reduce the angle of curvature or even to try to straighten the spine (this often happens in the case of younger patients aged 6–9).

According to Zaina, the use of high-quality conservative treatment has an impact on reducing the number of surgical procedures. Scoliosis-specific physiotherapy exercises (PSSEs) can reduce spinal deformities and improve quality of life as an isolated approach or in combination with bracing. The effectiveness of bracing depends on the appropriate selection of the brace, taking into account the Cobb angle, skeletal maturity, and hours per day of brace wearing [11].

According to our observations, the presence of parents/caregivers can be beneficial for the better understanding of the exercise itself and understanding of the purpose of the therapy. What is more, children’s motivation increased when parents/caregivers were present. This is why we were interested in the effect of parents’ assistance on FITS effectiveness. The main objective of this study is to compare the effectiveness of the conservative treatment of idiopathic scoliosis performed with the assistance of a parent or caregiver during a 5-day inpatient program compared to 2-week inpatient program without parents. The authors’ experience indicates that the parents’ presence and involvement in the therapy process significantly improves the therapeutic effects and enables precise performance of exercises at home. Moreover, the parents’ participation took into account the age of the children. To the best of our knowledge, no such study has been published in the literature to date.

## 2. Materials and Methods

### 2.1. Study Design

A retrospective comparative study of two groups of adolescents with a similar pattern of idiopathic scoliosis (double-curve type), treated using the FITS method by the same team of physiotherapists, followed one protocol. The only difference was parental assistance. Group I comprised a 5-day program with parental assistance, and Group II comprised a 2-week program without parental assistance. The tests were performed at the beginning of the first day and at the end of the last day of the stay.

### 2.2. Participants

The study involved 208 girls aged 10–17 with diagnosed double-curve idiopathic scoliosis, divided into two groups: Group I (5-day program) and Group II (2-week program).

### 2.3. Group I: 5-Day Program with Parental Assistance

Group I (5-day program with parental assistance) consisted of 119 girls with an average age of 13.3 years (SD = 1.8). The average Cobb angle was 29.3° (SD = 10.6) in the thoracic spine and 29° (SD = 9.6) in the lumbar spine, with a mean Risser score of 2 (SD = 1.9), with 68%. The angle of trunk rotation (ATR) at the thoracic and lumbar prominences averaged 7.2° (SD = 4) and 5° (SD = 4.2), respectively. Scoliosis larger than 30° was observed in 63% of participants, and 97 children (81.5%) used bracing. Pre-treatment values included an Anterior Trunk Symmetry Index (ATSI) of 26.6 (SD = 12.5), a Posterior Trunk Symmetry Index (POTSI) of 23.7 (SD = 12), and a Posterior Trunk Symmetry Index in Correction (POTSI COR) of 22.8 (SD = 11.7).

### 2.4. Group II: 2-Week Program Without Parental Assistance

Group II (2-week program without parental assistance) consisted of 89 girls with an average age of 13.9 years (SD = 1.8). The average Cobb angle was 31.8° (SD = 12.5) in the thoracic spine and 28.4° (SD = 10.6) in the lumbar spine, with a mean Risser score of 2.6 (SD = 1.9), with menarche 77.5%. The angle of trunk rotation at the thoracic and lumbar prominences averaged 7.3° (SD = 3.9) and 4.7° (SD = 4.4), respectively. Scoliosis larger than 30° was observed in 59.5% of participants, and 74 children (83.2%) used bracing. Pre-treatment values included an ATSI of 26.6 (SD = 10.8), a POTSI of 24.1 (SD = 11.4), and a POTSI COR of 22.4 (SD = 11.1).

### 2.5. Intervention

Children in Group I participated in the program with parents or caregivers, whose participation was mandatory. Parents assisted their children during the exercises; supported the explanation of the exercises, helping the children understand them better; controlled the quality of performance; and motivated them to make an effort. What is more, they supported the children emotionally with their presence. Moreover, they recorded the exercises for them to do independently. They gained a deeper understanding of their child’s specific scoliosis type and self-correction strategies and received guidance on continuing these practices at home. Children in Group II participated in the program alone, performing all exercises without parental assistance or supervision. The exercises they learned during the program were video-recorded along with the corresponding corrective movement patterns and self-corrective instructions to enable participants to continue practicing them at home. Both programs included specific physiotherapy exercises according to the Functional Individual Treatment of Scoliosis Method (FITS). The primary objective was to enhance sensory-motor performance, improve the shift of movement toward the corrected side, achieve derotation to correct curvature, refine movement patterns in supine and sitting positions, practice derotational breathing both with and without bracing, stabilize the lower trunk, maintain self-correction, and integrate corrected posture as much as possible into activities of daily living. All participants in each group performed the same types of following exercises: preparation of the soft tissue to corrective movement, correction patterns, breathing exercising, stabilization in 3D correction, and self-corrective movement. Corrective patterns were tailored to each individual’s curvature characteristics.

### 2.6. Outcome Measures

Photographic registration was employed as an objective, reliable, reproducible, and non-invasive method for evaluating body posture. This approach focused on three parameters: ATSI, POTSI, and POTSI COR. The procedure strictly adhered to established standards, including study setup, subject preparation, and photographic registration protocols [12,13,14]. It was used to evaluate the child’s posture at the beginning of the program and to analyze the potential for improving both habitual and self-corrected posture.

The following bony landmarks were marked on the participants’ bodies with a black non-toxic marker: central notch in the sternum for ATSI and the spinous process of the seventh cervical vertebra (C7) for POTSI. Children were also asked to tie up their hair to ensure that the landmarks were clearly visible. The photographs were taken by using a Fujifilm x-T10 camera equipped with a Stroboss 36 flash, against a posturography grid as the background according to the standards developed by Stoliński [13]. Participants stood on a marked cross, and photographs were taken at the beginning and end of the program. Images were captured in habitual posture (with the face or back facing the camera) and in corrected posture (with the back facing the camera).

**Figure 1:**
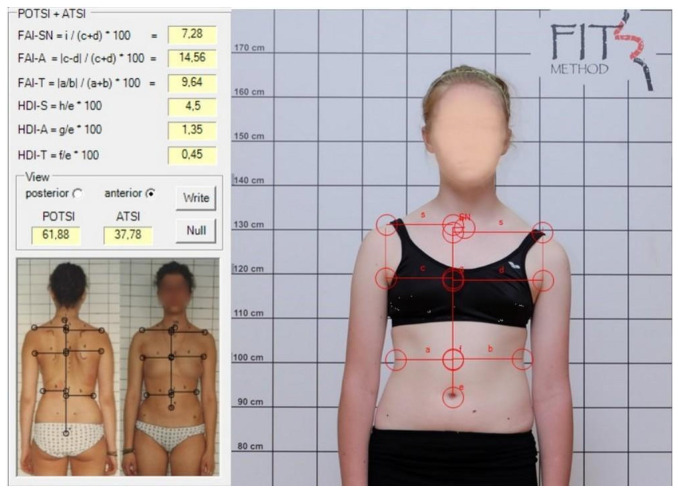
ATSI measurement in spontaneous position.

The photographs were analyzed on a computer using SCODIAC 2.6 software, which calculated trunk symmetry indices, ATSI and POTSI [13]. After manually marking specific landmarks on the photographs in SCODIAC, the indices were automatically calculated. For ATSI, the following landmarks were identified: the jugular notch of the manubrium; the central point of the umbilicus; and bilaterally a point on the shoulder where the horizontal shoulder line intersects the vertical line extending from the axillary fold, the most prominent point of the anterior axillary fold, and the deepest waist indentation (Figure 1).

**Figure 2:**
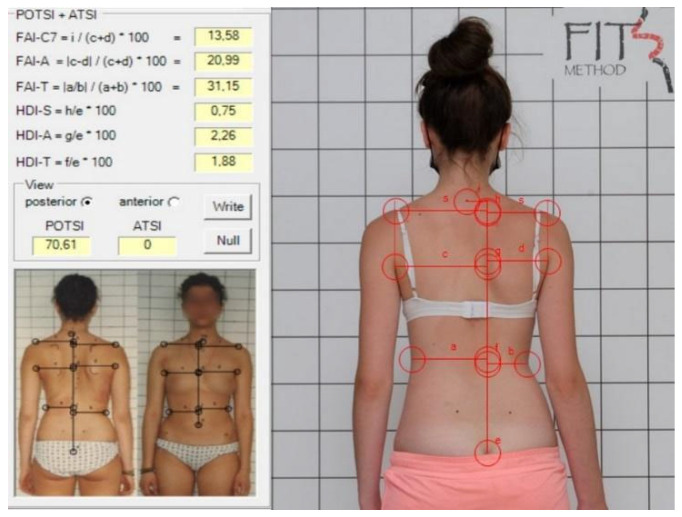
POTSI measurement in a spontaneous position.

**Figure 3:**
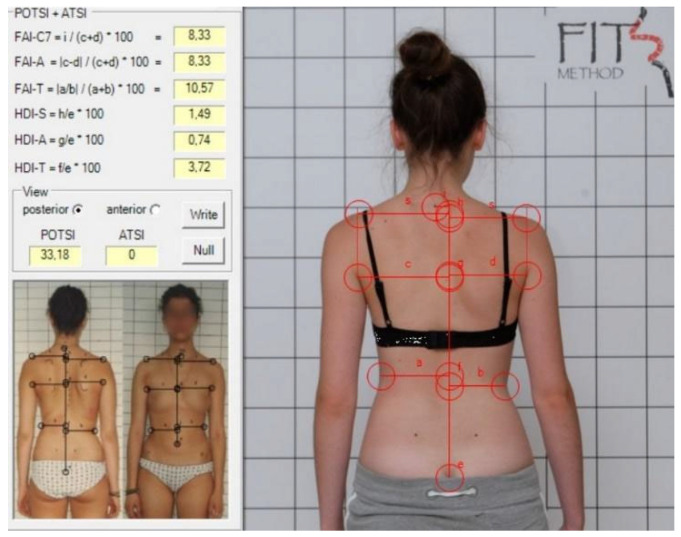
POTSI COR measurement in a corrected position.

For POTSI and POTSI COR, the following landmarks were identified: spinous process of C7; the top part of the gluteal cleft; and bilaterally a point on the shoulder where the horizontal shoulder line intersects the vertical line extending from the axillary fold, the most prominent point of the posterior axillary fold, and the deepest waist indentation (Figure 2 and Figure 3) [13].

Objective evaluation of postural changes in children, conducted before and after treatment programs, can be performed using standardized photographic methods and precise landmark analysis. A lower value of the ATSI and POTSI indices indicates better body symmetry [14]. Moreover, the outcomes of the clinical examination before and after the treatment program have been compared. This examination included the projection of the vertical from the external occipital protuberance onto the gluteal cleft and the distance to the peak of the curvatures and to the shoulder blades from the vertical line. Additionally, the angle of trunk rotation was measured using a scoliometer in the Adams test, and it is analyzed in this article. The comparison of those outcomes was also the determinant of the patients’ clinical improvement.

### 2.7. Statistical Methods

Statistical analysis of the collected data was conducted using StatSoft’s Statistica 10.3 software. Non-parametric tests were employed to analyze the variables, because the parameters did not follow a normal distribution, as confirmed by the Shapiro–Wilk test. The Mann–Whitney U Test was used to assess differences in the average values between the two populations. The Wilcoxon Signed-Rank Test was applied to evaluate changes within the same group. The Spearman rank correlation coefficient was used to determine the correlation between two variables that did not meet normality assumptions. The chi-square test was used for analyzing categorical variables. A *p*-value of 0.05 was considered statistically significant.

### 2.8. Ethical Considerations

The study was conducted in accordance with the Declaration of Helsinki and approved by the Ethics Committee of the Poznań University of Medical Sciences (number—212/24) on 6 March 2024 for studies involving humans. Patient confidentiality and data protection were ensured according to current international regulations [15]. Informed consent was obtained from participants and their parents/caregivers.

## 3. Results

**Figure 4:**
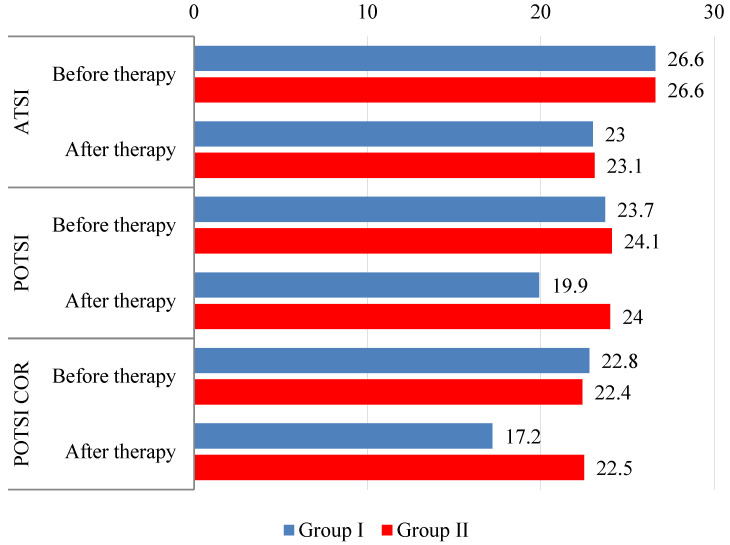
ATSI, POTSI, and POTSI COR values before and after therapy according to Table 1, Table 2 and Table 3.

The difference in Anterior Trunk Symmetry Index (ATSI) scores before and after treatment was not significant between the groups (*p* = 0.967). However, post-treatment measures within each group showed statistically significant differences. In Group I, 68.9% of patients achieved better (lower) values after therapy (*p* < 0.001). The average improvement in this group was 7.4 (improvement more than 5–56.25%). In Group II, 66.3% of patients achieved better results after therapy (*p* = 0.001). The average improvement in this group was 8.9 (improvement more than 5–59.02%), (Table 1, Figure 4).

The difference in Posterior Trunk Symmetry Index (POTSI) scores in habitual posture before treatment was not statistically significant between the groups (*p* = 0.688). Post-treatment measures revealed a statistically significant improvement only in Group I (*p* < 0.001)—lower values were observed in 64.7% of the patients. The average improvement was 9 (improvement more than 5–63, 79%) (Table 2, Figure 4).

In corrected posture, the values of the Posterior Trunk Symmetry Index in Correction (POTSI COR) after treatment compared to pre-treatment were statistically significant only in Group I (*p* < 0.001), whereas in Group II the change was insignificant (*p* = 0.380). Significant differences between the groups were measured after treatment (*p* < 0.001); therefore, the treatment effect was significant in Group I (*p* < 0.001). Lower values after therapy were observed in 77.3% of the patients. The average improvement was 9.9 (improvement more than 5–71.01%) (Table 3, Figure 4).

No significant correlation was found in Group I (Table 4). A statistically significant correlation between ATSI and POTSI values was observed pre- and post-treatment in Group II—higher ATSI values corresponded with higher POTSI values (Table 5).

The angle of trunk rotation (ATR) values measured with the Bunnell scoliometer placed in the thoracic region over the most prominent point of the curvature showed a significant difference before and after treatment in both groups: Group I *p* < 0.001 and Group II *p* = 0.007. The degree of improvement in the two groups was not statistically significant (*p =* 0.093) (Table 6).

In the lumbar region, the ATR value showed a statistically significant difference after treatment compared to pre-treatment in Group I (*p =* 0.026) only. The degree of improvement in the groups did not show a statistically significant difference (*p =* 0.388) (Table 7).

## 4. Discussion

The idea for this study emerged during the COVID-19 pandemic. During the period of restrictions, the possibility of providing two-week therapy was limited. Additionally, children had to be accompanied by their parents or caregivers. Soon, we realized that the therapy outcomes were superior to those of the previous therapy model. Previously, 2-week inpatient programs were organized (2002–2020). Five-day inpatient programs have been organized since 2020.

Physiotherapeutic scoliosis-specific exercises (PSSEs) demonstrated efficacy in the management of idiopathic scoliosis (IS). However, ensuring adolescents’ compliance and adherence to the physiotherapy protocol remains a challenge. Active parental participation could contribute to mitigating this limitation. The use of specific methods, including Functional Individual Treatment of Scoliosis (FITS), in managing idiopathic scoliosis in children aims not only to halt the progression of the condition but also to reduce the curvature angle (spinal deformity) and improve trunk appearance. These efforts ultimately contribute to enhancing the children’s overall and mental well-being, which are key factors in improving their quality of life [1]. The appearance can be assessed either subjectively or objectively (surface topography, photogrammetry, or photographic registration).

Photogrammetry involves the precise measurement of three-dimensional objects based on two-dimensional photographs [16,17]. However, it is often not validated [18]. The literature provides examples of various protocols for setting up registration equipment, with the camera typically positioned 3 m from the object. There are variations in camera height relative to the object, ranging from 70–90 cm or half of the object’s height [19,20,21,22]. Some authors do not specify the camera height [18]. One such photogrammetric method, the Moire technique, positions the camera 2.6 m from the object at a height equal to half of the patient’s back length [17]. The photographic registration used in this study follows a standardized protocol, as outlined by the authors [13].

Children with scoliosis tend to evaluate their posture from the front, as this is what they see in the mirror. In contrast, parents often assess posture from the front as well as from the back, as this is where asymmetries in the shoulder girdle, scapulae, waist triangles, and pelvic girdle are more clearly visible. Parents are often alarmed by the appearance of prominent areas on the trunk, such as a thoracic or lumbar hump. However, both the child’s and the parent’s assessments are subjective. Therefore, we were interested in the possibility of a correlation between ATSI and POTSI.

The trunk symmetry assessment method by means of photographic registration was developed by Dr. Suzuki in 1999, who described it as a reliable measure for evaluating trunk appearance in patients with scoliosis [12]. To evaluate the Anterior Trunk Symmetry Index (ATSI) and Posterior Trunk Symmetry Index (POTSI) in the studied scoliosis group, results established by other authors should be used as reference. Innami conducted a study involving 55 healthy children and 155 children with scoliosis, who had an average Cobb angle of 22.4° (ranging from 10° to 57°) and a mean age of 13.8 years. The obtained POTSI values were 16.5 (SD = 8.2) for healthy children and 28.1 (SD = 12.8) for children with scoliosis [22]. The value of POTSI for children with scoliosis is higher than the results obtained in this study: 23.74 in Group I (SD = 11.97) and 24.05 (SD = 11.44) in Group II (Table 2).

Yagci also reported higher baseline POTSI values in a smaller group of children (30 subjects) aged 12 years and older, with scoliosis angles ranging from 20° to 45°. These children were treated using the Scientific Exercises Approach to Scoliosis (SEAS) method, and their POTSI values decreased significantly from 33.7 to 20.7 [23]. The high improvement rates in POTSI were attributed to the fact that the study subjects had not received any prior treatment. From the start of treatment, the children used bracing, and the treatment lasted for 4 months. The group treated with the FITS method was diverse regarding prior exercise and bracing history. A significant difference between the SEAS and FITS approaches was the duration of therapy (4 months versus 5 days, respectively).

Results similar to those obtained using the FITS method were reported by Changov [24]. In a group of 72 subjects with two-curve scoliosis, the ATSI and POTSI indices were measured before and after a 5-day therapy based on the Schroth method. On average, the ATSI and POTSI indices improved by 3.1 and 3.5, respectively.

Stoliński et al. reported an average ATSI value of 25.3 in healthy children aged 7–10 years [14]. Matlęga provided values for younger healthy children (average age 7.1 years), with POTSI at 28.45 and ATSI at 35.54 [25]. However, those groups consisted of children under 10 years of age, i.e., not included in this study. Kotwicki et al. assessed 105 girls aged 13.7 ± 1.9 years (54 of them with a Cheneau brace). In girls with scoliosis of 25–40 degrees, POTSI was 25.5 ± 16.7 (treated with a brace) and 24.5 ± 11.5 (without a brace). In scoliosis above 45°, POTSI was 33.4 ± 17.5 (treated with a brace) and 41.0 ± 22.1 in the non-brace group, respectively [26]. Durmała et al. divided older patients (the average age was 20.7 ± 1.88) into two groups: Group I—scoliosis below 30° (19 children) and Group II—more than 30° (17 children). All children were braced and subjected to intensive inpatient exercises according to the Dobomed method. The group with a lower value of the Cobb angle received an average POTSI result on the level of 20.55 ± 12.33, whereas Group II had 28.21 ± 16.03 [27].

The authors of this article, in a manner similar to Matlęga and Changov, observed higher ATSI values compared to POTSI values. However, poorer posture is typically more apparent from the back than from the front, as clinical evaluation of trunk asymmetry is not performed from the front, and no standardized method for such evaluation has been developed. The assessment should be expanded to include analysis in the sagittal and transverse planes, allowing for a more complete, objective, and three-dimensional evaluation of posture. The authors of this article highlight the absence of standardized protocols for the sagittal, transverse, and coronal planes (ATSI and POTSI) that could be used to evaluate healthy children aged 10 years and older, i.e., during the period of peak growth spurt and highest risk of scoliosis progression. Establishing such standards could enable more accurate analysis of the obtained results.

The detected improvement in the measured parameters was small but statistically significant. The clinical significance was observed as a clear improvement of the trunk symmetry by the parents, patients, and therapists.

It is difficult to compare the results quoted above with the results obtained by the authors of this article because the number of relevant publications concerning parental participation in physiotherapy to enhance compliance and efficacy is limited.

### 4.1. Study Limitations

This study has several limitations: its retrospective design, potential variations in participants’ prior exercise or bracing history, the inability to provide long-term follow-up data, not including the analysis of one- and three-curved scoliosis, and short time of observation. Also, small differences between groups in age (6 months) or Risser (0.6 point) could potentially contribute to the worse outcomes in Group I.

### 4.2. Clinical Implications and Future Perspectives

In our opinion the findings of this study may influence future clinical practice and patient care. The FITS method is continually refined, resulting in improved therapy quality, enhanced effectiveness of therapeutic techniques, and better recommended corrective patterns. The 5-day program using the FITS method demonstrated greater effectiveness compared to the 2-week program, suggesting that parental presence and involvement in the therapeutic process significantly enhance adherence and motivation. Continuous parental participation helps the child learn the exercises more accurately and continue practicing at home, while also enhancing both the child’s and parent’s sense of security and motivation as a team. During the stationary program the parents can experience the exercises their child performs, gaining insight into the effort and concentration required to achieve the goal. At the same time, parents also become responsible for the treatment process. The above studies expose the need for larger parental involvement, extending the length of time parents spend with their child and their ability to supervise the exercises performed at home. Then a child feels more cared for and motivated and moreover appreciates the parent’s help.

Analyzing the above observations and based on the reports of Romano et al., it would be worthwhile to extend these observations until the end of the treatment period to make these observations more reliable [28].

## 5. Conclusions

1. Functional Individual Therapy of Scoliosis (FITS method) applied as 5-day inpatient therapy and including parental participation improved ATSI, POTSI, and ATR parameters of children with idiopathic scoliosis.
2. Parental involvement and active participation in the treatment program enhance therapy outcomes in a short period of time while equipping parents with the skills needed to continue practicing the exercises accurately at home.

## Figures and Tables

**Table 1:** Anterior Trunk Symmetry Index (ATSI) values before and after therapy.

ATSI	Group I	Group II	*p*
Before therapy	26.6 ± 12.5	26.6 ± 10.8	*p* = 0.691
After therapy	23.0 ± 10.0	23.1 ± 10.3	*p* = 0.864
Difference	3.6 ± 8.1	3.5 ± 9.4	*p* = 0.967
*p* before-after	***p* < 0.001**	***p* = 0.001**	

**Table 2:** Posterior Trunk Symmetry Index (POTSI) values before and after therapy.

POTSI	Group I	Group II	*p*
Before therapy	23.7 ± 12.0	24.1 ± 11.4	*p* = 0.688
After therapy	19.9 ± 9.7	24.0 ± 12.0	***p* = 0.012**
Difference	3.8 ± 8.5	0.1 ± 7.7	***p* = 0.005**
*p* before-after	***p* < 0.001**	*p* = 0.419	

**Table 3:** Posterior Trunk Symmetry Index in Correction (POTSI COR) values before and after therapy.

POTSI COR	Group I	Group II	*p*
Before therapy	22.8 ± 11.7	22.4 ± 11.1	*p* = 0.689
After therapy	17.2 ± 7.8	22.5 ± 10.4	***p* < 0.001**
Difference	5.6 ± 9.4	0.1 ± 9.7	***p* < 0.001**
*p* before-after	***p* < 0.001**	*p* = 0.380	

**Table 4:** Analysis of the relationship between ATSI and POTSI before and after therapy in Group I (Spearman’s rank correlation coefficient).

Parameters	R	*p*
26.6 (ATSI before) vs. 23.7 (POTSI before)	0.08	0.409
23.0 (ATSI) after vs. 19.9 (POTSI after)	0.11	0.246

**Table 5:** Analysis of the relationship between ATSI and POTSI before and after therapy in Group II (Spearman’s rank correlation coefficient).

Parameters	R	*p*
26.6 (ATSI before) vs. 24.1 (POTSI before)	**0.31**	**0.003**
23.1 (ATSI after) vs. 24.0 (POTSI after)	**0.26**	**0.015**

**Table 6:** ATR value in thoracic scoliosis.

ATR Th	Group I	Group II	*p*
Before therapy	7.2 ± 4.0	7.3 ± 3.9	*p* = 0.905
After therapy	6.1± 3.7	6.8 ± 3.9	*p* = 0.361
Difference	1.1 ± 1.9	0.5 ± 2.2	*p* = 0.093
*p* before-after	***p* < 0.001**	***p* = 0.007**	

**Table 7:** ATR value in lumbar scoliosis.

ATR L	Group I	Group II	*p*
Before therapy	5.0 ± 4.2	4.7 ± 4.4	*p* = 0.410
After therapy	4.6 ± 3.8	4.4 ± 3.7	*p* = 0.623
Difference	0.4 ± 2.0	0.3 ± 2.4	*p* = 0.388
*p* before-after	***p* = 0.026**	*p* = 0.281	

## Data Availability

The data presented in this study are available on request from the corresponding author due to legal and ethical reasons.

## References

[1] Negrini S., Donzelli S., Aulisa A.G., Czaprowski D., Schreiber S., de Mauroy J.C., Diers H., Grivas T.B., Knott P., Kotwicki T. (2018). 2016 SOSORT guidelines: Orthopaedic and rehabilitation treatment of idiopathic scoliosis during growth. Scoliosis Spinal Disord..

[2] Stokes I.A., Burwell R.G., Dangerfield P.H. (2006). Biomechanical spinal growth modulation and progressive adolescent scoliosis-a test of the ‘vicious cycle’ pathogenetic hypothesis: Summary of an electronic focus group debate of the IBSE. Scoliosis.

[3] Bettany-Saltikov J., Parent E., Romano M., Villagrasa M., Negrini S. (2014). Physiotherapeutic scoliosis—Specific exercises for adolescents with idiopathic scoliosis. Eur. J. Phys. Rehabil. Med..

[4] Seleviciene V., Cesnaviciute A., Strukcinskiene B., Marcinowicz L., Strazdiene N., Genowska A. (2022). Physiotherapeutic Scoliosis-Specific Exercise Methodologies Used for Conservative Treatment of Adolescent Idiopathic Scoliosis, and Their Effectiveness: An Extended Literature Review of Current Research and Practice. Int. J. Environ. Res. Public Health.

[5] Ma K., Wang C., Huang Y., Wang Y., Li D., He G. (2023). The effects of physiotherapeutic scoliosis-specific exercise on idiopathic scoliosis in children and adolescents: A systematic review and meta-analysis. Physiotherapy.

[6] Negrini S., Donzelli S., Negrini A., Parzini S., Romano M., Zaina F. (2019). Specific exercises reduce the need for bracing in adolescents with idiopathic scoliosis: A practical clinical trial. Ann. Phys. Rehabil. Med..

[7] Schreiber S., Whibley D., Somers E. (2023). Schroth Physiotherapeutic Scoliosis-Specific Exercise (PSSE) Trials-Systematic Review of Methods and Recommendations for Future Research. Children.

[8] Berdishevsky H., Lebel V.A., Bettany-Saltikov J., Rigo M., Lebel A., Hennes A., Romano M., Białek M., M’hango A., Betts T. (2016). Physiotherapy scoliosis—Specific exercises—A comprehensive review of seven major schools. Scoliosis Spinal Disord..

[9] Białek M. (2011). Conservative treatment of idiopathic scoliosis according to FITS concept: Presentation of the method and preliminary, short term radiological and clinical results based on SOSORT and SRS criteria. Scoliosis.

[10] Białek M., M’hango A., Bettany-Saltikov J., Paz-Lourido B. (2012). FITS Method. Physical Therapy Perspectives in the 21st Century—Challenges and Possibilities.

[11] Zaina F., Donzelli S., Negrini S. (2023). Idiopathic Scoliosis: Novel Challenges for Researchers and Clinicians. Children.

[12] Suzuki N., Inami K., Ono T., Kohno K., Asher M.A. (1999). Analysis of Posterior Trunk Symmetry Index (POTSI) in scoliosis, Part 1. Studies in Health Technology and Informatics.

[13] Stolinski L., Kozinoga M., Czaprowski D., Tyrakowski M., Cerny P., Suzuki N., Kotwicki T. (2017). Two-dimensional digital photography for child body posture evaluation: Standardized technique, reliable parameters and normative data for age 7–10. Scoliosis Spinal Disord..

[14] Stoliński Ł., Czeszejko-Sochacka E., Czaprowski D. (2022). Wykorzystanie fotorejestracji w obiektywizacji oceny postawy ciała. Prakt. Fizjoterapia I Rehabil..

[15] Official Journal of the European Union (2016). Regulation (EU) 2016/679 of the European Parliament and of the Council of 27 April 2016 on the Protection of Natural Persons with Regard to the Processing of Personal Data and on the Free Movement of Such Data, and Repealing Directive 95/46/EC (General Data Protection Regulation).

[16] Musak J. (2020). Fotogrametryczna ocena postawy ciała osób ze skoliozą. Państwo I Społeczeństwo.

[17] Barczyk K., Hawrylak A., Wojna D., Giemza C. (2008). Zastosowanie fotogrametrii komputerowej do oceny wybranych parametrów postawy ciała dzieci w młodszym wieku szkolnym. Acta Bio-Opt. Inform. Medica.

[18] Dilian O., Kimmel R., Tezmah-Shahar R., Agmon M. (2022). Can We Quantify Aging-Associated Postural Changes Using Photogrammetry? A Systematic Review. Sensors.

[19] Santos M.M., Silva M.P.C., Sanada L.S., Alves C.R.J. (2009). Photogrammetric postural analysis on healthy seven to ten-year-old children: Interrater reliability. Rev. Bras. Fisioter..

[20] Souza J.A., Pasinato F., Basso D., Castilhos Rodrigues Correa E., Toniolo da Silva A.M. (2011). Biophotogrammetry: Reliability of measurements obtained with a posture assessment software (SAPO). Rev. Bras. Cineantropom. Desempenho. Hum..

[21] Milanesi J.M., Borin G., Corre’a E.C.R., da Silva A.M.T., Bortoluzzi D.C., Souza J.A. (2011). Impact of the mouth breathing occurred during childhood in the adult age: Biophotogrammetric postural analysis. Int. J. Pediatr. Otorhinolaryngol..

[22] Inami K., Suzuki N., Ono T., Yamashita Y., Kohno K., Morisue H. (1999). Analysis of Posterior Trunk Symmetry Index (POTSI) in Scoliosis, Part 2. Studies in Health Technology and Informatics.

[23] Yagci G., Yakut Y. (2019). Core stabilization exercises versus scoliosis—Specific exercises in moderate idiopathic scoliosis treatment. Prosthet. Orthot. Int..

[24] Chongov B. (2017). Correlation between scoliosis deformity type and trunk asymmetry before and after Schroth physiotherapeutic exercises. Medicine.

[25] Matlęga A., Stępowska J., Wiśniewski A., Gajewski J. (2020). Assessment of the coronal plane trunk symmetry in children. Physiother. Theory Pract..

[26] Kotwicki T., Kinel E., Chowańska J., Bodnar-Nanuś A. (2008). POTSI, Hump Sum and Sum of Rotation—New surface topography parameters for evaluation of scoliotic deformity of the trunk. Pol. J. Physiother..

[27] Durmała J., Blicharska I., Drosdzol-Cop A., Skrzypulec-Plinta V. (2015). The Level of Self-Esteem and Sexual Functioning in Women with Idiopathic Scoliosis: A Preliminary Study. Int. J. Environ. Res. Public Health.

[28] Romano M., Minozzi S., Bettany-Saltikov J., Zaina F., Chockalingam N., Kotwicki T., Maier-Hennes A., Arienti C., Negrini S. (2024). Therapeutic exercises for idiopathic scoliosis in adolescents. Cochrane Database Syst. Rev..

